# Hepatoprotective effect of meal replacement seeds juice based on sweet potato (MRSJ) against CCl_4_-induced cytotoxicity in HepG2 cells

**DOI:** 10.1007/s10068-019-00709-2

**Published:** 2019-11-28

**Authors:** Hye-Jung Park, Song Min Lee, Hee Sook Kim, Ji-Youn Kim, Sang-Hyeon Lee, Jeong Su Jang, Mun Hyon Lee

**Affiliations:** 1 Food Research Center, Angel Co., Ltd., Busan, 46988 Korea; 2 Major in Pharmaceutical Engineering, Division of Bioindustry, College of Medical and Life Sciences, Silla University, Busan, 46958 Korea

**Keywords:** Hepatoprotection, Liver function, Meal replacement juice, Sweet potato, Antioxidant

## Abstract

A sweet potato-based Meal Replacement Seeds Juice (MRSJ) was developed by mixing sweet potatoes and carrots with four types of seeds. Consuming the MRSJ rather than the whole vegetables or whole seeds improved digestive function, proving that it is suitable for the elderly. Its rich composition of minerals, vitamins, and unsaturated fatty acids implicates it as an excellent nutrient source. Notably, the ethyl acetate fraction of MRSJ contains abundant phenolics. The antioxidant activity assays showed that these phenolics have high radical scavenging activity, reducing power, and antioxidant capacity similar to l-ascorbic acid. The ethyl acetate fraction exerted protective effects against CCl_4_- or H_2_O_2_-induced hepatotoxicity via DNA protection, lipid accumulation inhibition, and cell protection, wherein ALT and AST activities in the cell culture solution decreased significantly. These findings suggest that MRSJ consumption may protect against liver diseases. Moreover, MRSJ as an excellent nutrient source may be developed as an age-neutral food.

## Introduction

The steep increase in the global aged population necessitates the development of a novel diet considering the physical and physiological characteristics of the elderly. The unique problems of aged people include decreased digestion and absorption, dental loss with increasing age, and reduced general muscle reflexes involved in swallowing. The intake of whole vegetables and whole grains is often difficult for older people to digest and absorb them, causing discomfort even after meals. Thus, the demand for the development of a Home Meal Replacement (HMR) that can be enhanced absorption of nutrition and enjoyed easily by both the elderly and busy modern people, is high.

The modern society has also features the global problem of higher incidence of various liver diseases caused by increased exposure to toxic substances due to smoking, alcohol consumption, and environmental pollution (Marcellin and Kutala, 2018). Liver plays a crucial role in the metabolism of major nutrients, energy homeostasis, metabolism of hormones, detoxification, and immune or antimicrobial actions. Various drugs based on natural substances have been developed for hepatic diseases. Generally, CCl_4_- or D-galactosamine-induced liver damage is used as a model for studies on developing hepato-protective or therapeutic drugs. CCl_4_ treatment leads to the production and activation of reactive intermediates including trichloromethyl radical (CCl_3_·) and its derivative, trichloromethylperoxy radical (CCl_3_OO·), via its metabolism mediated by cytochrome P_450_ (CYP2E1) in liver microsome (Dai et al., 2014). The generated free radicals cause lipid hyperoxidation by attacking the lipid membranes in the vicinity, induce hepatotoxicity involving necrosis, and promote fatty infiltration or reduced microsomal enzyme activity by binding to the macromolecules such as intracellular proteins and lipids. They can change the antioxidant status of the tissues including the release of cytosolic enzymes, such as alanine aminotransferase (ALT), aspartate aminotransferase (AST), alkaline phosphatase (ALP), and lactate dehydrogenase (LDH), into the serum, and ultimately mediate apoptosis (Shen et al., 2015). Hence, the removal of ROS is critical in the treatment and prevention of hepatic diseases. Thus, regarding liver damage as the major cause of chronic diseases in modern society, this study aimed to develop a functional meal replacement that can fulfill multiple requirements, such as: (a) convenient to use by the elderly and busy modern people, (b) useful in maintaining liver health, and (c) able to prevent and treat hepatic diseases. For this, a Meal Replacement Seeds Juice (MRSJ) was prepared by mixing four types of seeds, which exhibit a diverse nutrients and functionality, with sweet potatoes (*Ipomoea batatas* L.), a popular ingredient in meal replacement, and carrots, a widely used ingredient in vegetable juices. The potential of MRSJ in the development of a functional meal replacement was evaluated with an aim to propose a novel form of food intake in the busy and aged modern society.

## Materials and methods

### Materials and sample pretreatment

Vegetables; sweet potato (*Ipomoea batatas* L.) and carrot (*Daucus carota*) and seeds; evening primrose seed (*Oenothera odorata*), job’s tear (*Coix lacryma*-*jobi*), oat (*Avena sativa*) and peanut (*Arachis hypogaea*) used in this experiment were purchased from a farmers’ market (Busan, Korea) in February 2018. The purchased vegetables and seeds were washed with water and then made into extracts using a lower-speed juice extractor at 82 rpm (Angelia 8000, Angel Co., Ltd., Busan, Korea).

### Chemicals

Gallic acid, naringin, 3-(4,5-dimethylthiazol-2-yl)-2,5-diphenyltetrazolium bromide (MTT), 2,2-diphenyl-1-picrylhydrazyl (DPPH), 2,2-azino-bis(3-ethylbenzothiazoline-6-sulfonic acid (ABTS), N-hippuryl-histidyl-leucine (HHL) and oil red O staining solution were supplied by Sigma-Aldrich (St. Louis, MO, USA). Dulbecco’s Modified Eagle’s Medium (DMEM) and Fetal Bovine Serum (FBS) were supplied by Hyclone Laboratories (Logan, UT, USA).

### Analysis of basic composition

The general content of MRSJ was analyzed in accordance with the Korean Food Standards Codex (MFDS, 2019). The moisture content was determined by a 105 °C atmospheric pressure heating and drying method, crude protein content by the micro-Kjeldahl method using an automatic nitrogen analyzer (Kjeltec Auto 2300, Foss, Hilleroed, Denmark), crude fat content by the acid hydrolysis method and crude ash content by the direct incineration method.

### Analysis of mineral content

Using a method described in the Korean Food Standards Codex, mineral content was analyzed by mixing 1 g of MRSJ and 10 mL of HNO_3_ solution, heating at 190 °C for 35 min in a microwave (Multiwave 3000, Anton Paar Gmbh, Graz, Austria) to completely remove the solvent and cooling the sample before analysis. Spectrophotometry was performed by ICP-OES (Optima 8300, PerkinElmer, Waltham, MA, USA) for other minerals and by ICP-MS (DRC-e, PerkinElmer, Waltham, MA, USA) for selenium.

### Analysis of vitamin content

Vitamin A and E were determined according to Korean Food Standards Codex (MFDS, 2019). Vitamin B in the filtrate was determined by modified Martins-Junior’s method (Martins-Junior et al., 2008). Vitamin compositions were analyzed via high-performance liquid chromatography (Agilent 1200, Agilent, Santa Clara, CA, USA).

### Analysis of fatty acid content

Fatty acid methyl esters (FAMEs) of MRSJ were prepared by Korean Food Standards Codex (MFDS, 2019) and analyzed using a gas chromatograph (GC-7890, Agilent, Santa Clara, CA, USA) with flame ionization detection.

### Determination of digestive enzyme activities in vegetables and seeds

The α-amylase activity of MRSJ was determined by a modified 3,5-dinitrosalicylic acid (DNS) method (Doehlert and Duke, 1983). Protease activity was determined using casein as the substrate (MFDS, 2019). To compare amylase and protease activity between whole vegetables, seeds and extracted juice, 1% starch agar plates and 1% skim milk plates were prepared. Water extracted samples were added to well on plate and incubated 37 °C for 24 h. The enzyme activity was evaluated by measuring the size of clearance sites.

### Improvement effect on digestion function of juice extraction

To investigate the effect of juice extraction on digestion, pure enzymes (α-amylase and protease) were added to 1 g of whole vegetables piece, seeds and extracted juices. The activities of each enzymes depending on extraction were measured using DNS and casein methods, as mentioned before.

### Lipid peroxidation assay

Screwcap tube containing a mixture of MRSJ (4.0 mL), 2.5% linoleic acid in ethanol (4.1 mL), 0.02 M sodium phosphate buffer (pH 7.0, 8.0 mL), and distilled water (3.9 mL) was kept at 40 °C in the dark. Subsequently, the degree of oxidation was measured using ferric thiocyanate method (Hisateru et al., 1966).

### Superoxide dismutase (SOD)-like activity assay

The SOD-like activity of MRSJ was determined using a commercially available SOD assay kit (Dojindo Molecular Technologies, Kumamoto, Japan).

### Solvent fraction

To evaluate physiological function of MRSJ, solvent fractionation was conducted. MRSJ methanol extract (100 g) was reconstituted in water (500 mL) and vigorously extracted by hexane (500 mL) in 2 L separating funnel. The solution was left for phase separation after extraction. The organic phage was withdrawn, and another 500 mL hexane was added into the remaining aqueous solution for extraction again. This fractionation process was repeated in triplicate. The fraction of hexane was combined and dried by a rotary evaporator. A similar fractionation process was also carried out to prepare dichloromethane, ethyl acetate and butanol fractions.

### Antioxidation activities

The DPPH radical scavenging activity was measured by modified Blois’s method (Blois, 1958). ABTS radical cation solution was diluted in distilled water to obtain an OD_734_ of 0.70 ± 0.01. The diluted solution was added to each MRSJ extracts. After 1 h incubation in the dark, the absorbance was measured at 734 nm. l-Ascorbic acid solution was used as the positive control group. The reducing antioxidant power was measured by the FRAP assay as described by Benzie and Strain, 1996.

### Analysis of total phenolic content (TPC) and total flavonoid content (TFC) content

The amounts of TPC and TFC, which are closely correlated with antioxidant activity, were measured as described by Folin and Denis (1912) and Davis (1947), respectively. The calibration curves of TPC and TFC were drawn using gallic acid and naringin, respectively.

### Analysis of DNA damage by hydroxyl radical using electrophoresis

The oxidation of DNA exposed to the hydroxyl radical created by Fenton’s reaction was performed in accordance with an existing protocol (Oyaizu, 1986). The DNA was resolved through 1% agarose gel at 100 V for 30 min. The gel was stained with Midori Green and observed under UV light using the DAVINCH-Chemi Imaging System (CAS-400SM, Corebiosystem, Seoul, Korea).

### Cell culture

HepG2 cells (American Type Culture Collection, Manassas, VA, USA) were cultured in the DMEM containing 10% fetal bovine serum, 2 mM glutamine and 100 µg/mL penicillin–streptomycin in an incubator (MCO-15AC, SANYO Electric Co., Ltd., Gunma, Japan) that was maintained at 5% CO_2_ and 95% or higher humidity at 37 °C.

### MTT assay

According to the method of Hansen et al. (1989), the cytotoxicity of HepG2 cells in MRSJ extracts was measured using the MTT assay.

### Hepatoprotective activity assay

HepG2 cells were plated in a 24-well plate for 24 h. Then, the medium was aspirated and replaced with serum-free medium with MRSJ extracts for 1 h and then the toxin solution containing CCl_4_ and DMSO, which were suspended in DMEM medium via ultrasonic dispersion, was treated for 10 h. After removing the supernatant of each well, the cytotoxicity was measured using the MTT assay.

### ALT and AST activity assay

ALT and AST activities in the supernatant after exposure of cells to 20 mM CCl_4_ for 6 h were determined using colorimetric assay kit (AST; #K753-100, ALT; #K752-100, Biovision Co., CA, USA).

### DNA ladder assay

For the DNA fragmentation analysis, the cells were treated with different concentrations of ethyl acetate fraction. DNA was extracted with phenol–chloroform–isoamyl alcohol (25:24:1) mixture. High molecular weight DNA was then pelleted at 13,000×*g* for 10 min at 4 °C, and the low molecular weight DNA in the supernatant was removed and precipitated overnight in two volumes of ice-cold ethanol at − 70 °C.

### Oil red O staining

After 24 h co-culture with 10 mM CCl_4_, HepG2 cells were washed two times with PBS. Cells were stained with Oil Red O staining solution for 10 min at 18–20 °C. The stained cells were imaged under a microscope.

### Statistical analysis

All analyses were performed at least 3 times and expressed as means and standard deviations (mean ± SD). For a significant difference in the mean, this study employed Duncan’s multiple comparisons in one-way analysis of variance by using SPSS (version 20.0, SPSS, Inc., Chicago, IL, USA). The mean between the two experimental groups was analyzed by Turkey’s multiple comparison test, and *p*-values of *p *< 0.05 were considered significant.

## Results and discussion

### MRSJ preparation and nutritional composition

The nutrient content of MRSJ showed highest content of moisture, characteristic of extracted juices, followed by carbohydrates, crude fat and crude protein (Table 1). Approximately 80% of the dry weight of the sweet potatoes is carbohydrates (Wang et al., 2016). Only a trace amount of fat is contained in sweet potatoes, and the reason why the high crude fat content after carbohydrate in MRSJ has been determined to be due to the addition of oil seeds with high fat content. Mineral content showed that potassium (328.23 mg/100 g) and phosphorous (123.42 mg/100 g) were the highest, followed by magnesium and calcium (Table 1). Furthermore, selenium, which is known to have efficacy against cancer, heart diseases, immune diseases and inflammatory diseases (Burk, 2002; Kieliszek and Błażejak, 2016), content in the MRSJ increased by approximately 3.48 fold from approximately 2.3 µg/100 g in sweet potatoes. Considering that the percentage of sweet potatoes in the MRSJ is 40%, MRSJ can take 8.70 fold higher selenium than a single sweet potato. This increase corresponds to 66.67% of the daily recommended intake for adult men (60 µg/day) from 500 mL of MRSJ a day. Among the plant-origin foods including green vegetables, root vegetables and fruits, excluding the animal-origin foods such as fish and meat, the seeds like cereals are known to have the highest selenium content (Morris and Levander, 1970; Slavin Joanne et al., 2001; Thorn et al., 1978). Slavin Joanne et al. (2001) reported that selenium and vitamin E contained in whole seeds exhibit synergistic effects with phytochemicals, antioxidants and phenolics in foods, which may protect against various diseases. Thus, the intake of sweet potato juice in combination with seeds further enhances nutritional values by supplementing the nutrients such as selenium that are deficient in root vegetables. Further, MRSJ contains high amount of vitamin E and β-carotene (Table 1). Vitamin E, which is at approximately 1.01 mg/100 g of sweet potatoes, increased to 4.49 mg/100 g (approximately 4.45 fold), probably due to the addition of oats that are enriched with vitamin E (Sterna et al., 2016) and oil seeds such as evening primrose seed. Considering that the percentage of sweet potatoes in the MRSJ is 40%, MRSJ can take 11.11 fold higher vitamin E than a single sweet potato. This corresponds to a content satisfying the daily recommended intake of 12 mg α-TE/day for adult men upon the intake of only 300 mL MRSJ. Fatty acid content in MRSJ showed that the ratio between saturated and unsaturated fatty acids was approximately 2:8 (data not shown). MRSJ contains abundant unsaturated fatty acids and the contents of oleic acid and linoleic acid were shown to be 1.52 mg/100 g and 1.54 mg/100 g, respectively, accounting for 36.67% and 37.08% of the total fatty acid content. The juice extraction of sweet potatoes and carrots with extremely low fat content, after the addition of oil seeds, has been determined to be the reason for the increased content of essential oils. Thus, by carrying out the juice extraction of sweet potatoes and carrots after the addition of seeds, the possibility of ensuring more abundant nutrient supply that may be deficient in root vegetables as several nutrients including selenium, essential oils and vitamin E was shown. These useful ingredients are known to have superior antioxidant activity and physiological functions, and based on this, the potential of MRSJ as a functional meal replacement for antioxidant and hepatoprotective effect was evaluated in this study.

**Table 1:** Nutrition composition of MRSJ

Composition	Unit	MRSJ
General composition		
Carbohydrate	g/100 g	20.26 ± 0.05
Crude protein	g/100 g	3.96 ± 0.01
Crude fat	g/100 g	4.58 ± 0.01
Crude ash	%	0.95 ± 0.02
Moisture	%	70.24 ± 0.03
Calorie	kcal/100 g	138.16 ± 0.02
Mineral		
Phosphorus	mg/100 g	123.42 ± 0.76
Magnesium	mg/100 g	62.72 ± 0.41
Calcium	mg/100 g	36.42 ± 0.86
Potassium	mg/100 g	328.23 ± 1.71
Zinc	mg/100 g	0.75 ± 0.01
Manganese	mg/100 g	1.12 ± 0.01
Iron	mg/100 g	1.15 ± 0.02
Sodium	mg/100 g	32.69 ± 1.63
Copper	mg/100 g	0.43 ± 0.04
Selenium	mg/kg	0.08 ± 0.02
Vitamin		
β-Carotene	mg/100 g	3.78 ± 0.02
Vitamin B1	mg/100 g	0.03 ± 0.00
Vitamin B2	mg/100 g	0.03 ± 0.00
Vitamin B3	mg/100 g	ND^a^
Vitamin B5	mg/100 g	ND
Vitamin B6	mg/100 g	0.09 ± 0.00
Vitamin E	mg/100 g	4.49 ± 0.06
Fatty acid		
Palmitoleic acid (C16:1)	g/100 g	0.005 ± 0.00
Oleic acid (C18:1)	g/100 g	1.523 ± 0.04
Linoleic acid (C18:2)	g/100 g	1.540 ± 0.04
Linolenic acid (C18:3n-3)	g/100 g	0.017 ± 0.00
Gadoleic acid (C20:1)	g/100 g	0.033 ± 0.00
Eicosadienoic acid (C20:2)	g/100 g	0.004 ± 0.00
Phenolic compounds		
Total phenolic contents	mg GAE/100 g	115.09 ± 0.18
Total flavonoid contents	mg NE/100 g	25.88 ± 0.02

Data are mean ± SD of at least three replicates of MRSJ

^a^*ND* not detected

### Effects of MRSJ on improving the digestive function

**Fig. 1:**
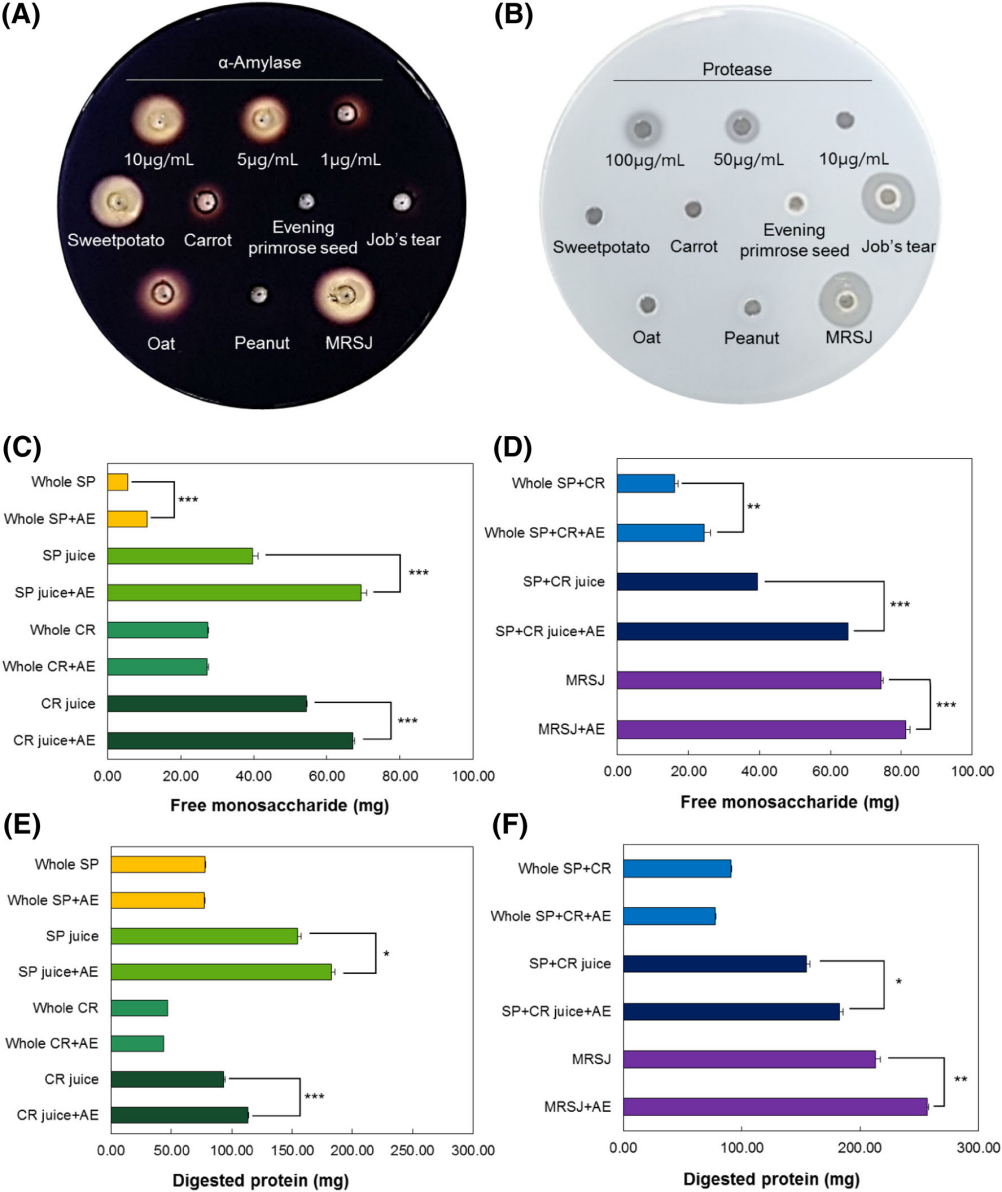
Effect of MRSJ on amylase activity and effect of juice extraction on improvement of digestive function. (**A**) Amylase and (**B**) protease activities of whole vegetables, whole seeds and MRSJ. (**C**, **D**) α-Amylase activities of whole vegetables, whole seeds and MRSJ. (**E**, **F**) Protease activities of whole vegetables, whole seeds and MRSJ. SP, sweet potato; AE, α-amylase enzyme; CR, carrot. Different letters indicated significant differences among samples. ***p < 0.001; **p < 0.01; *p < 0.05

To examine whether digestion is being assisted after the intake of MRSJ, the activities of α-amylase and protease were determined. The result showed that α-amylase activity was 36.45 unit/g, while protease activity for hydrolyzing protein was 31.28 unit/g, indicating high activities of digestive enzyme (data not shown). In addition, when the amylase activity was determined on the starch plate by loading each of the four seeds and sweet potatoes and carrots independently (Fig. 1A), the activity was detected in sweet potatoes, carrot and oats as independent materials. For sweet potatoes was showed strong the starch hydrolysis level compare to 10 µg/mL pure amylase enzyme used as the positive control. However, MRSJ that has been prepared using the juice extraction after mixing each individual material, showed a higher level of starch hydrolysis than sweet potato independent material. The enhanced ability to decompose starch is thought to be due to synergistic effects among individual materials in the mixture. To evaluate the protease activity each of independent materials and MRSJ were loaded to skim milk plate (Fig. 1B). As a result, MRSJ was showed higher proteolysis activity than pure protease 100 µg/mL and other materials was not confirmed on proteolysis activity except for job’s tear. In previous study, by mixing carrots with whole buckwheat, evening primrose seed, sesame seed and safflower seed, it was shown that the intake of food materials in combination leads to marked increase in not only nutritional value but also in the activities of amylase, protease and antioxidants (Park et al., 2017). Further, examining the effects of juice extraction on the improvement of digestive function, the carbohydrate and protein decomposition activities of digestive enzymes before and after the juice extraction were determined. As shown in Fig. 1C, when the extracted juice of sweet potato was treated with amylase enzyme, free monosaccharides was 29.67 mg, showing a 5.73-fold higher level of production than sweet potato pieces. This has been determined to be the result of far easier digestion of the extracted juice than the pieces of sweet potato without extraction, when the treatment used an identical concentration of amylase enzyme. This suggests that the hydrolysis by amylase is far more active in the state of extracted juice. When sweet potato and carrot pieces were mixed by 0.5 g each to make up 1 g in total, the amylase enzyme treatment led to the production of 8.45 mg free monosaccharides (Fig. 1D), whereas in the state of extracted juice, free monosaccharides reached 25.58 mg, indicating 3.03 fold higher amylase enzyme activity. When the four seeds were added, the addition of seeds was shown to have slightly reduced the digestive enzyme activity; nevertheless, even without the addition of digestive enzymes, the inherent high level of free monosaccharides in MRSJ is likely to allow easy absorption of nutrients purely by the ingestion itself. Next, the effects on improving protein hydrolysis based on juice extraction was determined (Fig. 1E, F). The result showed that, when 1 g of sweet potato and carrot pieces were treated with protease enzyme, protein hydrolysis was almost negligible. However, treating the extracted juice with protease enzyme led to free amino acids of 27.59 mg in sweet potatoes and 19.58 mg in carrots, showing that the enzyme activity increased when the juice was treated with the enzyme. Moreover, although hydrolysis by protease enzyme did not occur in the pieces of sweet potato and carrot, protein hydrolysis was detected in the juice containing a mixture of sweet potatoes and carrots as 8.44 mg free amino acids were produced. In addition, 43.73 mg free amino acids were produced in MRSJ where the four seeds had been added to the juice extraction, showing an increased enzyme activity. Such findings may indicate that digesting the juice obtained through crushing and extraction is far easier than when whole vegetables or seeds that are ingested. Thus, MRSJ is proposed as a suitable candidate for an HMR that can be taken without any burden by the elderly who show reduced functions of mastication and digestion.

### Antioxidant activities of MRSJ and the change of total phenolic content and antioxidant capacity by solvent fractionation

**Fig. 2:**
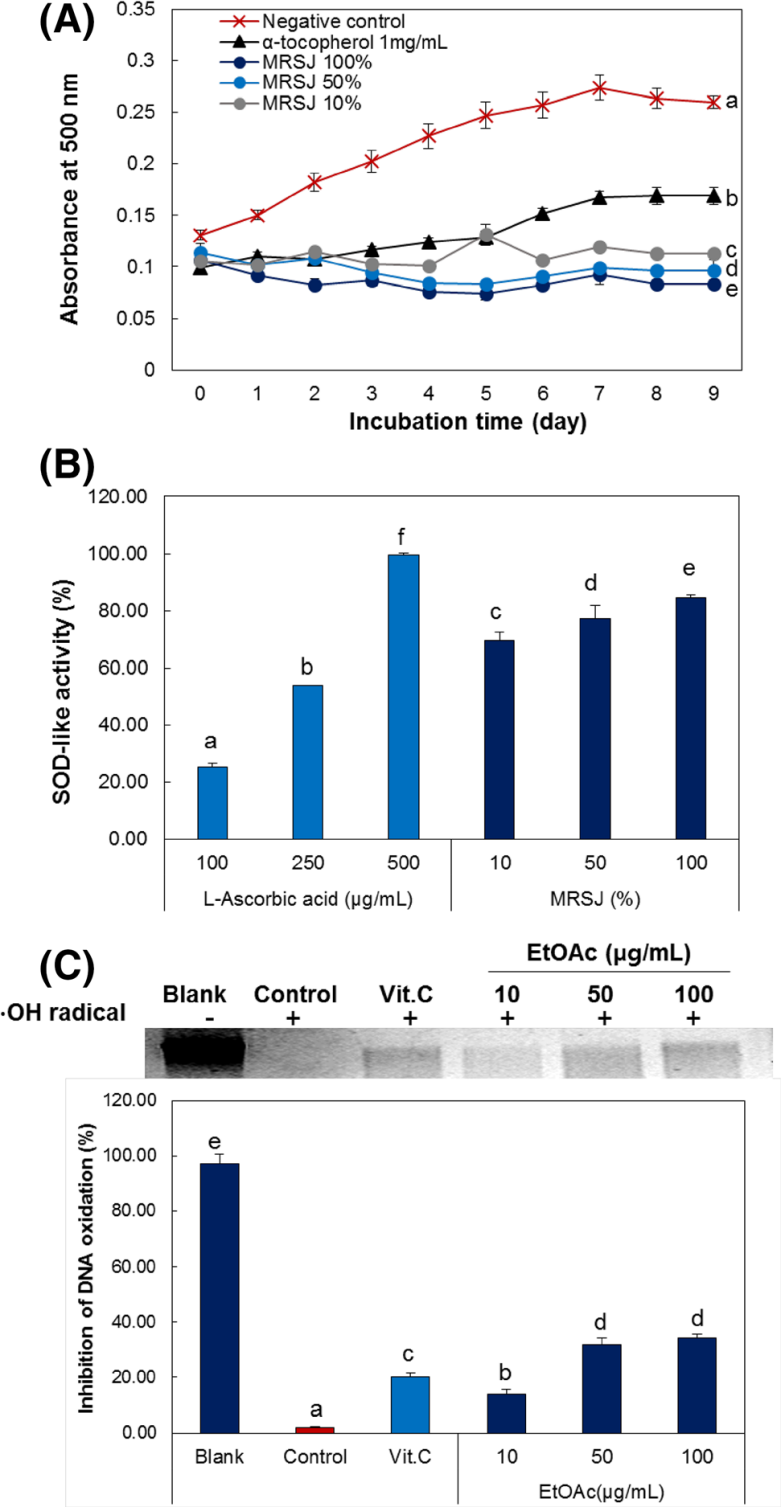
Antioxidative effect of MRSJ and EtOAc fractions. (**A**) Inhibition activity on lipid peroxidation and (**B**) SOD-like activity of MRSJ. (**C**) DNA protective effect against Fenton’s reaction 20 mM CCl_4_ of EtOAc fraction. Different letters indicated significant differences at *p *< 0.05 among samples

To evaluate antioxidant activities of MRSJ, lipid peroxidation and SOD-like activity assay were conducted. First of all the inhibitory effect of lipid peroxidation according to the treatment of the MRSJ methanol extract (1:4) was measured using the thiocyanate method by incubation of linoleic acid at 40 °C for 9 days (Fig. 2A). As the incubation time increased by 7 days, the increase in absorbance was observed due to formation of Fe^3+^-thiocyanate complex with lipid oxidation. On the other hand, MRSJ methanol extract (1:4) showed a 95.56% inhibitory rate of lipid peroxidation at 10% dilution. Next, the result of SOD-like activity of MRSJ methanol extract was indicated at Fig. 2B. The SOD-like activity by MRSJ methanol extract was increased to 84.63% as the concentration increased. This was higher than the 250 μg/mL of l-ascorbic acid used as a positive control. Therefore, it is indicated that MRSJ can significantly eliminate reactive oxygen species produced in the body and the oxidative damage to the cell structure due to their high antioxidant activity for lipid peroxidation and SOD-like activity. Next, to assess the functionality of MRSJ, solvent fractions of the MRSJ-methanol extract were obtained using each of the following six solvents: hexane (9.08%), dichloromethane (1.60%), ethyl acetate (0.53%), butanol (9.29%), and water (79.49%). The total phenolic content was 7.10 mg/g in the MRSJ-methanol extract, and among the six solvent fractions of methanol extract, a substantially high phenolic content was exhibited by the ethyl acetate fraction (180.01 mg/g). Most phenolics are observe in ethyl acetate and butanol fractions, but it is also reported that there are very few phenolics and flavonoids in the fat soluble fractions such as hexane and dichloromethane (Mariod et al., 2009; Termentzi et al., 2008). The representative polyphenols contained in sweet potatoes are caffeic acid, cyanidins, peonidins, quercetin, tiliroside, astragalin, rhamnocitrin, rhamnetin and kaempferol (Mohanraj and Sivasankar, 2014). Due to the abundance of these components, sweet potatoes exhibit antioxidant capacity of 42.94% on vitamin C (Mohanraj and Sivasankar, 2014). In fact, the antioxidant activity measured for each fraction (Table 2) showed that the DPPH and ABTS radical scavenging activities were also markedly higher in the ethyl acetate fraction, where the total phenolic and flavonoid contents were equally far higher. The IC_50_ value for the DPPH radical scavenging activity in the ethyl acetate fraction was 0.09 mg/mL, which did not deviate greatly from that of l-ascorbic acid (IC_50_ value = 0.02 mg/mL) used as the positive control. On the contrary, for the ABTS radical scavenging activity, the IC_50_ value in the ethyl acetate fraction was 0.34 mg/mL, compared to 0.05 mg/mL for l-ascorbic acid, indicating that the radical scavenging activity in ethyl acetate fraction was slightly less than that of l-ascorbic acid. Nonetheless, compared to that in the five other fractions, the radical scavenging activity in the ethyl acetate fraction was distinctly higher, which is attributed to the rich contents of phenolic compounds and other useful components in the ethyl acetate layer. Next, MRSJ was assessed for the reducing power (Table 2). The reducing ability of the ethyl acetate fraction (1.13 mM FeSO_4_/g) was almost identical to that of the positive control l-ascorbic acid (1.24 mM FeSO_4_/g). These results are in agreement with the those of studies on sweet potatoes exhibiting high DPPH and ABTS radical scavenging activities, lipid peroxidation inhibitory activity, and reducing power (Wang et al., 2016). Next, inhibitory effect on DNA oxidation by hydroxyl radical was investigated for ethyl acetate fraction indicating the highest antioxidant activity. When Fenton’s reaction was used to expose the genomic DNA to hydroxyl radicals (Fig. 2C), the oxidative damage of DNA was shown to have led to the degradation of most DNA and in the case of treatment with MRSJ ethyl acetate fraction, a high level of DNA protective effects was confirmed. This indicated the strong antioxidant activity of MRSJ based on the similar pattern of activity to the positive control l-ascorbic acid. These results are in agreement with the reports on the protective effects of various enzymes in sweet potatoes against the DNA damage induced by hydroxyl radicals (Wang et al., 2016). These results suggest that MRSJ, owing to the phenolic compounds and flavonoids in addition to the high content of essential oils, vitamins and minerals such as selenium, exhibits excellent radical scavenging activity and reducing power, and by significantly reducing accumulation of harmful ROS levels in the body, may provide protection against oxidative damage.

**Table 2:** Phenolic contents and antioxidant activities of solvent fractions of MRSJ

Fractions	Radical scavenging activity (IC_50_)		Reducing power (FRAP)	Total phenolic contents	Total flavonoid contents
	DPPH	ABTS			
l-Ascorbic acid	0.02 ± 0.00 mg/mL^a^	0.05 ± 0.00 mg/mL^a^	1235.95 ± 0.00 µM FeSO_4_/g^d^	–	–
MeOH extracts	4.12 ± 0.40 mg/mL^b^	10.34 ± 0.32 mg/mL^e^	102.40 ± 1.33 µM FeSO_4_/g^a^	7.10 ± 0.13 mg GAE/g^a^	2.15 ± 0.41 mg NE/g^b^
Hexane	1.59 ± 0.02 mg/mL^ab^	6.49 ± 0.12 mg/mL^d^	202.41 ± 4.82 µM FeSO_4_/g^b^	8.06 ± 0.18 mg GAE/g^a^	8.40 ± 0.72 mg NE/g^c^
CH_2_Cl_2_	9.30 ± 0.80 mg/mL^c^	14.47 ± 0.37 mg/mL^f^	35.01 ± 0.32 µM FeSO_4_/g^a^	5.91 ± 0.07 mg GAE/g^a^	0.68 ± 0.33 mg NE/g^a^
EtOAc	0.09 ± 0.02 mg/mL^a^	0.34 ± 0.11 mg/mL^b^	1129.98 ± 1.35 µM FeSO_4_/g^c^	180.01 ± 9.08 mg GAE/g^c^	28.19 ± 1.43 mg NE/g^d^
BuOH	1.06 ± 0.12 mg/mL^ab^	4.47 ± 0.29 mg/mL^c^	294.14 ± 10.56 µM FeSO_4_/g^c^	15.64 ± 0.70 mg GAE/g^b^	7.86 ± 0.40 mg NE/g^c^
Water	16.83 ± 3.69 mg/mL^d^	21.71 ± 0.09 mg/mL^g^	31.62 ± 1.60 µM FeSO_4_/g^a^	5.28 ± 0.04 mg GAE/g^a^	0.81 ± 0.01 mg NE/g^a^

Data are mean ± SD of at least three replicates of each samples

### Cytoprotective effects of ethyl acetate and butanol fractions on CCl_4_ or H_2_O_2_ induced HepG2 cell damage

**Fig. 3:**
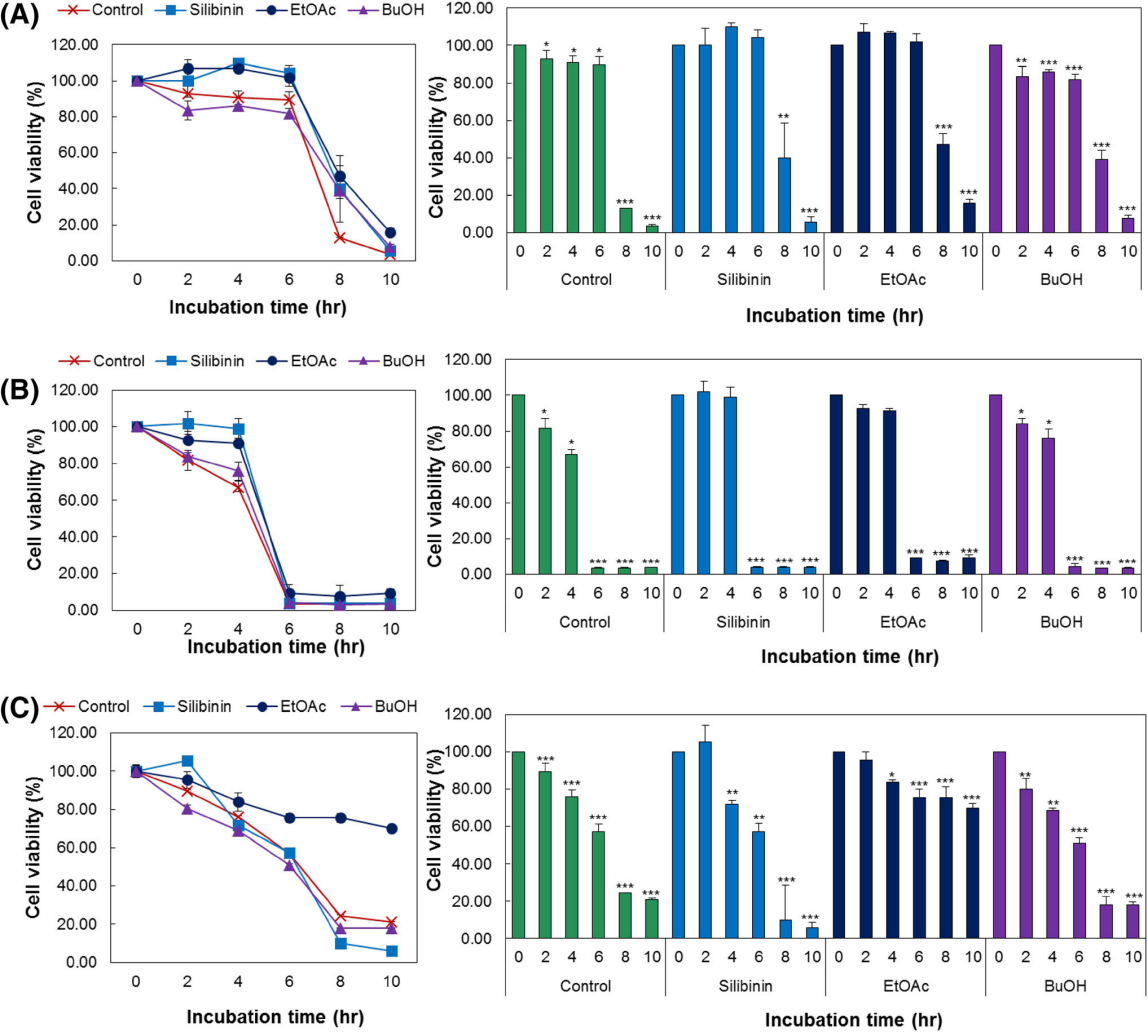
Hepatoprotective effect of EtOAc and BuOH fraction of MRSJ on HepG2 cell damage. Hepatoprotective effect on HepG2 cells against (**A**) 20 mM CCl_4_, (**B**) 30 mM CCl_4_ and (**C**) 10 mM H_2_O_2_. Different letters indicated significant differences among samples. ***p < 0.001; **p < 0.01; *p < 0.05

Human hepatoma (HepG2) cells were treated with CCl_4_ and H_2_O_2_ independently to induce liver cell damage and estimated subsequent cytoprotective effects of ethyl acetate and butanol fractions. Treatment of HepG2 cells with 20 mM CCl_4_ (Fig. 3A) resulted in a steady cytotoxicity from 2 h after the treatment and rapid apoptosis occurred after 8 h. The cells treated with silibinin showed no CCl_4_ induced cytotoxicity up to 6 h. After 8 h of CCl_4_ treatment, the silibinin-treated cells showed 39.9% viability, compared to 12.96% viability shown by the control, which indicated relatively high cytoprotective effects. In the presence of MRSJ, CCl_4_ induced cytotoxicity was not observed up to 6 h in the ethyl acetate fraction, which indicated cytoprotective effects and after 8 h of CCl_4_ treatment, cell viability was 47.27%, indicating stronger protective effects than in the case of silibinin-treated cells. Contrastingly, the butanol fraction showed CCl_4_ induced cytotoxicity after only 2 h of treatment as in the control, but absence of rapid increase in cytotoxicity after 8 h of treatment indicated cytoprotective effects. A similar trend of cytoprotective effects was observed when HepG2 cells that were treated with higher concentration of CCl_4_ at 30 mM (Fig. 3B). Purple flesh sweet potatoes show protection against liver cell damage induced by CCl_4_, due to the rich content of anthocyanins that acts as an antioxidant agent in preventing the oxidative damage by CCl_4_, thereby inhibiting lipid peroxidation and enhancing the activities of antioxidant enzymes such as superoxide dismutase and glutathione peroxidase (Wang et al., 2016). In contrast to the various reports on the hepatoprotective effects of purple flesh sweet potatoes, no study has yet reported on the effects of white, yellow or orange flesh sweet potatoes. Thus, the superior hepatoprotective effect of MRSJ prepared based on orange flesh pumpkin-sweet potatoes have been determined to be originating from synergistic combination with four seeds. As a result similar to this, Jung et al. (2015) reported that the crude extracts of purple flesh sweet potatoes exhibited stronger hepatoprotective effects than the purified anthocyanins, which was attributed to the additive or synergistic effects with other useful components contained in sweet potatoes. Therefore, although there is no hepatoprotective effect derived from anthocyanins, orange flesh-sweet potatoes can show hepatoprotective effect through synergistic combination with other seeds and carrot. Next, the MRSJ ethyl acetate fraction was examined for the cytoprotective effects against H_2_O_2_ induced HepG2 cell damage (Fig. 3C). When HepG2 cells were time-dependently treated with 10 mM H_2_O_2_ to induce cytotoxicity (Fig. 3C), the cells began to lose viability from 2 h of treatment, and after 6 h, the cell viability was shown to fall rapidly to 57.10%. Silibinin, the positive control, did not exhibit significant effects against the cytotoxicity induced by H_2_O_2_; however, the MRSJ ethyl acetate fraction exhibited higher protective effects than in the case of cytotoxicity induced by CCl_4_. The hepatoprotective effects of MRSJ ethyl acetate fraction were stronger against the harmful ROS produced during the treatment by H_2_O_2_ rather than by CCl_4_. This has been determined to be related to the outstanding antioxidant activity exhibited by the ethyl acetate fraction. Thus, the hepatoprotective effects of the MRSJ ethyl acetate fraction may be attributed to the outstanding antioxidant activity based on radical scavenging activity and reducing power of minerals such as selenium, vitamins, essential oils and phenolic compounds contained abundantly in MRSJ, whereby the hepatotoxicity of varying causes and the resulting shift in antioxidant state of the damaged liver cells are regulated.

### Cytoprotective effects of ethyl acetate fraction based on the assays of cytosolic enzyme activity, apoptosis, and lipid accumulation inhibitory activity

**Fig. 4:**
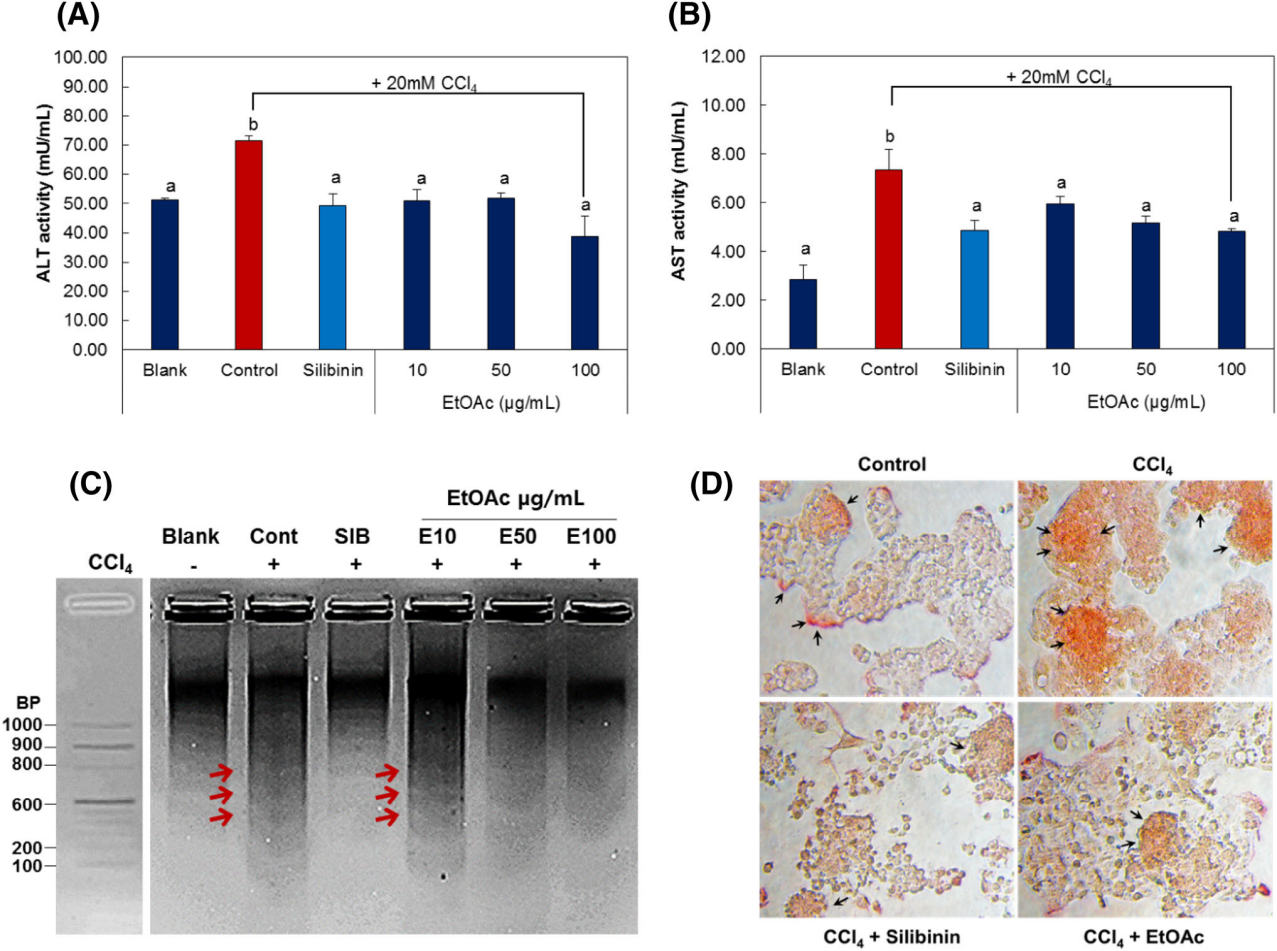
Inhibitory activities on liver health of EtOAc fraction of MRSJ. (**A**) ALT and (**B**) AST activities released from HepG2 cells. (**C**) Anti-apoptosis activity of MRSJ by DNA ladder assay. (**D**) Inhibition activities of MRSJ on lipid accumulation of HepG2. Significantly different at *p *< 0.05; different letters indicated significant differences among samples

Liver is an organ with markedly high concentration of enzymes and is connected to the blood circulation that allows easy release of the enzymes into the blood stream. This means that, when the liver cells are damaged upon CCl_4_ induced hepatotoxicity, cytosolic enzymes including ALT, AST, ALP and LDH are released into the serum (Shen et al., 2015). Thus, measuring the activity of enzymes such as ALT and AST is one of the most useful methods in hepatotoxicity study, and high enzyme activity is detected when the enzymes are released into the blood stream upon cell damage during the progression of liver cell degeneration and necrosis and the destruction of liver tissue. Hence, HepG2 cells were treated with 20 mM CCl_4_ to induce cytotoxicity and by determining ALT and AST activities, the cytoprotective effects of the ethyl acetate fraction were examined (Fig. 4A, B). As a result, the treatment with CCl_4_ was shown to significantly increase the activities of ALT and AST in HepG2 cell culture solution, while the activities were shown to decrease in the cell culture solution following the treatment with silibinin, the positive control and MRSJ ethyl acetate fraction. This has been determined to be due to the reduced secretion of cytosolic enzymes when CCl_4_ induced liver cell degeneration, necrosis and destruction were significantly inhibited. Notably, the ethyl acetate fraction led to the lower activities of ALT and AST than in the case of silibinin, indicating outstanding hepatoprotective effects. In addition, when the DNA of HepG2 cells damaged by CCl_4_ was examined (Fig. 4C), a distinct DNA ladder-like pattern was observed in comparison to the untreated blank. However, upon the treatment with silibinin as the positive control, the DNA ladder-like pattern was shown to have markedly reduced. Moreover, the treatment with increasing concentration of ethyl acetate fraction was also shown to have significantly reduced the DNA ladder-like pattern. The result indicates strong protective effects against the DNA damage induced by CCl_4_, which agrees with the cytoprotective effects of the ethyl acetate fraction. Furthermore, a characteristic of the toxicity induced by CCl_4_ is the rapid accumulation of triglycerides (TGs) in the liver as in the condition of steatotic liver tissue in patients with hepatic disease (Dai et al., 2014). In fact, when HepG2 cells are treated with CCl_4_, an excessive accumulation of adipocytes in the liver was observed (Fig. 4F). However, treating the cells with silibinin or ethyl acetate reduces the level of lipid accumulation to the level similar to the blank.

Therefore, this study has verified the rich contents of digestive enzymes in MRSJ, a type of seeds juice based on sweet potatoes prepared using a juice extractor with twin gear. Further, the MRSJ has far higher rate of digestion than whole vegetables or whole seeds due to the digestive enzymes and is suitable for a HMR for the elderly with reduced functions of mastication and digestion. Furthermore, seeds such as cereals and oil seeds are rich in selenium, vitamin E and unsaturated fatty acids that may be deficient in root vegetables, and therefore, MRSJ is an excellent nutrient source as it can supply the deficient nutrients. Moreover, by containing an abundance of useful components such as selenium, vitamins, essential oils and phenolic compounds, MRSJ exhibited radical scavenging activity and reducing power at a level similar to that exhibited by l-ascorbic acid. Based on such antioxidant activity, MRSJ also exhibited a substantially high level of hepatoprotective effects against CCl_4_ or H_2_O_2_ induced hepatotoxicity by DNA protection, inhibition of lipid accumulation and cell damage protection. These results thus suggest that MRSJ may protect against liver disease, a chronic condition in modern people who are exposed to high levels of air pollution and stress. Moreover, MRSJ as an excellent nutrient source enriched with minerals, vitamins and unsaturated fatty acids, may also contribute to the development of an age-neutral, senior-friendly food.

## References

[CR1] Benzie IF, Strain JJ (1996). The ferric reducing ability of plasma (FRAP) as a measure of “antioxidant power”: the FRAP assay. Anal. Biochem..

[CR2] Blois MS (1958). Antioxidant determinations by the use of a stable free radical. Nature.

[CR3] Burk RF (2002). Selenium, an antioxidant nutrient. Nutr. Clin. Care.

[CR4] Dai N, Zou Y, Zhu L, Wang H-F, Dai M-G (2014). Antioxidant properties of proanthocyanidins attenuate carbon tetrachloride (CCL_4_)–induced steatosis and liver injury in rats via CYP2E1 regulation. J. Med. Food.

[CR5] Davis W (1947). Determination of flavanones in citrus fruits. Anal. Chem..

[CR6] Doehlert DC, Duke SH (1983). Specific determination of α-amylase activity in crude plant extracts containing β-amylase. Plant Physiol..

[CR7] Folin O, Denis W (1912). On phosphotungstic-phosphomolybdic compounds as color reagents. J. Biol. Chem..

[CR8] Hansen MB, Nielsen SE, Berg K (1989). Re-examination and further development of a precise and rapid dye method for measuring cell growth/cell kill. J. Immunol. Methods.

[CR9] Hisateru M, Kyoden Y, Kimikazu I (1966). Antioxidative action of indole compounds during the autoxidation of linoleic acid. Eiyo to Shokuryo..

[CR10] Jung SB, Shin JH, Kim JY, Kwon O (2015). Shinzami Korean purple-fleshed sweet potato extract prevents ischaemia-reperfusion-induced liver damage in rats. J. Sci. Food Agric..

[CR11] Kieliszek M, Błażejak S (2016). Current knowledge on the importance of selenium in food for living organisms: a review. Molecules.

[CR12] Marcellin P, Kutala BK (2018). Liver diseases: A major, neglected global public health problem requiring urgent actions and large-scale screening. Liver Int..

[CR13] Mariod AA, Ibrahim RM, Ismail M, Ismail N (2009). Antioxidant activity and phenolic content of phenolic rich fractions obtained from black cumin (*Nigella sativa*) seedcake. Food Chem..

[CR14] Martins-Junior HA, Wang AY, Alabourda J, Pires MA, Vega OB, Lebre DT (2008). A chromatography-tandem mass spectrometry. J. Brazil. Chem. Soc..

[CR15] MFDS (Korean Ministry of Food and Drug Safety). Korean Food Standards Codex. Available from: https://www.foodsafetykorea.go.kr/foodcode/01_01.jsp. Accessed Jan. 9, 2019.

[CR16] Mohanraj R, Sivasankar S. Sweet Potato (*Ipomoea batatas* [L.] Lam)-A valuable medicinal food: A review. J. Med. Food 17: 733-741 (2014) 10.1089/jmf.2013.2818 24921903

[CR17] Morris V, Levander O (1970). Selenium content of foods. The Journal of nutrition.

[CR18] Oyaizu M. Studies on products of browning reaction: antioxidant activity of products of browning reaction. Jpn. J. Nutr. 44 (1986)

[CR19] Park S-H, Park H-J, Kim J-Y, Lee S-H, Jang JS, Lee MH (2017). Mixed seeds juice with high antioxidant capacity and digestive enzyme activity and its application. Food Sci. Biotechnol..

[CR20] Shen T, Li X, Hu W, Zhang L, Xu X, Wu H, Ji L (2015). Hepatoprotective effect of phenylethanoid glycosides from Incarvillea compacta against CCl_4_-induced cytotoxicity in HepG2 cells. J. Korean Soc. Appl. Biol. Chem..

[CR21] Slavin Joanne L, Jacobs D, Marquart LEN, Wiemer K (2001). The role of whole grains in disease prevention. J. Am. Diet. Assoc..

[CR22] Sterna V, Zute S, Brunava L (2016). Oat grain composition and its nutrition benefice. Agriculture and agricultural science procedia.

[CR23] Termentzi A, Kefalas P, Kokkalou E (2008). LC-DAD-MS (ESI+) analysis of the phenolic content of *Sorbus domestica* fruits in relation to their maturity stage. Food Chem..

[CR24] Thorn J, Robertson J, Buss DH, Bunton NG. Trace nutrients. Selenium in British food. J. Med. Food 39: 391-397 (1978) 10.1079/bjn19780049 629926

[CR26] Wang S, Nie S, Zhu F (2016). Chemical constituents and health effects of sweet potato. Food Res. Int..

